# A method to investigate the anti-metabolic activity of anti-cancer agents on ovarian cancer cells cultured in a 96-well high throughput format

**DOI:** 10.1186/s13048-015-0172-0

**Published:** 2015-07-04

**Authors:** Simon J. Hogg, John J. Evans, Peter H. Sykes, Kenny Chitcholtan

**Affiliations:** Peter MacCallum Cancer Centre, St Andrews Place, East Melbourne, 3002 VIC Australia; Department of Obstetrics and Gynaecology, Centre of Neuroendocrinology and MacDiarmid Institute for Advanced Materials and Nanotechnology, University of Otago, Christchurch, 2 Riccarton Avenue, Christchurch, 8011 New Zealand; Department of Obstetrics and Gynaecology, University of Otago, Christchurch, 2 Riccarton Avenue, Christchurch, 8011 New Zealand; Department of Obstetrics and Gynaecology, University of Otago, Level 3, Christchurch Women’s Hospital, Christchurch, 2 Riccarton Avenue, Christchurch, 8011 New Zealand

**Keywords:** Collagen gel, Ovarian cancer, Drug screening, Cisplatin

## Abstract

**Background:**

An early step of advanced ovarian cancer begins when floating cancerous cells as single cells or small clusters grow on the peritoneal surface. This surface is rich in extracellular matrix (ECM) proteins, which have profound effects on cellular behaviour and can facilitate cancer progression. Subsequently, this ECM may alter cellular metabolism making cancer cells susceptible to chemotherapeutic agents differently. Therefore, generating a cell culture tool in vitro that includes the interaction between ECM and cancer cells will facilitate our understanding of how cancer cells behave during cancer treatment. There is some evidence to suggest that in an in vitro model that includes ECM components such as collagens will provide a better predictive tool for drug evaluation than a traditional cell monolayer (2D) culture model.

**Findings:**

As a proof -of- concept, we made a collagen gel in a 96-well plate format and utilised this to evaluate the efficacy of clinical cytotoxic drugs, a targeted drug, and food compounds in single and combination treatments. The primary endpoints were to measure the reduction of cellular metabolism and secretion of vascular endothelial growth factor (VEGF). The invasive capacity of cancer cells was observed in collagen gels and it was cell line-dependent. The responses to drugs were prominently observed in collagen gels, but they had little effect on 2D cell monolayers. These responses were cell line- and type of drug-dependent.

**Conclusions:**

The collagen gel in a 96 well plate format was easy to set up and could have potential to identify drug sensitivity in the clinical management of women with platinum resistant ovarian cancer.

## Introduction

Ovarian cancer is the fourth most common cause of cancer related death for women in the developed world. It is usually advanced at diagnosis and chemotherapy normally prolongs life but is not curative. The high mortality of ovarian cancer is largely explained by the fact that patients present with widely metastatic disease within the peritoneal cavity [1]. Although most ovarian cancer initially responds well to the current front line cytotoxic agents, chemo-resistant recurrent disease commonly evolves and is usually fatal. Treatment is further complicated by the high degree of patient-to-patient tumour heterogeneity. Consequently, there is an urgent need for new therapeutic strategies and methods to enable variations in responses by distinct tumours to drugs to be studied. Further, the ability to study the effects of drug combinations, such as cytotoxic agents and other anticancer drugs (targeted agents or bioactive food compounds) has not been fully evaluated in ovarian cancer. To advance these goals, pre-drug screening tools that take account of some aspects of the tumour microenvironment should be developed.

Advanced ovarian cancer cells preferentially adhere and grow on top of the mesothelial lining of abdominal cavity (peritoneum). The peritoneum is rich in extracellular matrix (ECM) proteins, which offers a favourable attachment site for cancer cells that facilitates survival and metastatic invasion [2]. Therefore, we develop a gel-based method in which cells may invade a microenvironment containing collagen proteins. Thereby the ability of cells to grow in a three dimension (3D) following invasion is provided. Many studies have reported recently that cancer cells cultured in cell monolayer (2D) and 3D models show distinctive patterns of behaviour due to differential gene and protein expressions [3–5]. The imbalance of drug development and clinical selection may be attributed partly to limitations in the understanding of the underlying biological processes and the lack of suitable assays. We investigate the concept that an assay method that incorporates a 3D component is practical. The bedside problems are illustrated, for example, by patients with platinum resistant ovarian cancer. There are already many therapeutic options, and this choice is increasing at a rapid pace, but for these patients’ response rates to individual drugs is likely to be low. We, therefore, aim to introduce a framework for a clinically useful in vitro sensitivity assay that may have potential to avoid the necessity of trialing toxic or expensive drugs in patients who may not respond.

In this study, we selected drugs on the basis of current clinical discussions. Cytotoxic agents including paclitaxel and carboplatin are widely used to treat ovarian cancers. Targeted agents show promising results in other types of tumours, but there is a limited clinical data for ovarian cancer. Ovarian cancer cells show a dysregulation of PI-3 K/Akt/mTOR pathway. Therefore, an agent that specifically compromises these pathways is of great interest. Everolimus has been reported to show antitumour activity in pre-clinical tumour xenografts in mice [6]. Resveratrol and (−)-epigallocatechin-3-gallate (EGCG) are bioactive natural food compounds, and they have been reported to exhibit anti-tumour activities in in vitro and in vivo animal models [7, 8]. However, the use of resveratrol and EGCG as potential therapeutic agents for cancer treatment in a proper randomised clinical trial is still undetermined.

To approximate the microenvironment of an early growth stage of metastatic ovarian cancer cells at the peritoneal membrane, we have developed a collagen gel model in a 96 well plate format and culture ovarian cancer cells on top of the collagen gel. Cellular metabolism as an indication of cell viability and the production of secreted VEGF are the primary endpoints after treatment with selective cytotoxic drugs, a targeted agent and active food compounds. As a comparison, assays were also performed on cells cultured in similar conditions in a 2D monolayer.

## Materials and methods

The human ovarian adenocarcinoma cell lines, SKOV-3 and OVCAR-5, were obtained from Dr Judith McKenzie, Haematology Research group, University of Otago, Christchurch, New Zealand. SKOV-3 and OVCAR-5 cells were maintained in MEM and DMEM medium, respectively (GIBCO®, Life Technologies, New Zealand) supplemented with 10 % fetal bovine serum (FBS) (GIBCO®, Life Technologies, New Zealand), PenStrep (GIBCO®, Life Technologies, New Zealand) at a working concentration of 100 units/ml penicillin and 100 μg/ml streptomycin, 2 mM glutaMAX™ (GIBCO®, Life Technologies, New Zealand), and 2 μg/ml Fungizone® (Life Technologies, New Zealand). The final concentration of glucose in the media is 5.5 mM. The supplemented media is henceforth referred to as working media. SKOV-3 and OVCAR-5 cells in the working media were maintained at 37 °C in a humidified 5 % CO_2_ atmosphere.

### Generation of cell culture using collagen I and Geltrex™ and 2D cell monolayers in a 96-well plate

Partly confluent cell monolayers of SKOV-3 and OVCAR-5 were washed with PBS pH 7.4 and incubated with 1X trypsin-EDTA (Life Technologies, New Zealand) for 20–30 min. Cells were collected by a centrifugation at 300 g for 5 min. The cultures of ovarian cancer cells on top of collagen gels are previously described [9, 10]. Briefly, cell pellets were re-suspended in the working media and cell counts was obtained with a haemocytometer. A 96-well plate was overlaid with 60 μl mixture of cold collagen I (1 mg/ml, Sigma, New Zealand) and Geltrex™ (2.5 mg/ml, Life Technologies, New Zealand) solution and allowed to polymerise at 37 °C in a humidified 5 % CO_2_ atmosphere for 30 min. Cells (5000 cells in final volume of 200 μl working media) were added to each well and allowed to grow on top of polymerised collagens for 6 days with replacement of fresh media every 2 days. For cell monolayers, cell numbers and culture condition were similar to the 3D culture except non coated 96-well plates were used.

### Fluorescent image of cells cultured on top and inside collagen gels

#### For cells on top of collagen gels

The mixture of collagen solution (200 μl) was added to a 48 well plate and allowed to polymerise at the similar condition as described above. Single cell suspension (100,000 cells) were added onto the polymerised collagen gel and cultured for 6 days with replacement of fresh media every 2 days. Cells grown on top of collagen gel were fixed with 4 % paraformaldehyde in PBS pH 7.4 for 60 min and then washed extensively with PBS pH 7.4. Cells were then stained with Texas Red-Phalloidin (Molecular Probe, Life Technology) overnight at 4 °C and followed the staining with 10 ug/ml Hoechst 33492 (Molecular Probe, Life Technology) for 20 min. Cells were washed extensive with ice cold PBS pH 7.4 plus 0.1 % Tween-20.

#### For cells inside collagen gels

A round coverslip (20 mm diameter, ProSciTech, Australia) was overlaid with 150 μl cold mixture of collagen type I and Geltrex™ at final concentrations 1 mg/ml and 2.5 mg/ml, respectively. Gels were allowed to polymerise at 37 °C in a humidified 5 % CO_2_ atmosphere for 20 min. Cells (100,000) were mixed with 100 μl cold collagen solution and added on the top of first polymerised collagen gel. The gels were allowed to polymerise at 37 °C in a humidified 5 % CO_2_ atmosphere for 20 min. Cover slips were then carefully placed in 12-well culture plate and 1.5 ml working media. Cells were maintained in this condition for 6 days with replacement of fresh media every 2 days. Cells inside collagen gels were fixed with −20 °C acetone:methanol (50 %:50 % vol:vol) for 20 min at 4 °C. Cells were washed with PBS pH 7.4 four times for 10 min each washing. Cells were blocked with 4 % BSA and incubated with anti-actin antibody (Santa Cruz, Biotechnology Inc, USA) overnight at 4 °C. Cells were then washed four times for 10 min each washing and incubated with a 1/500 dilution of a secondary antibody conjugated with FITC for 90 min at 37 °C. Cells were then stained with 10 μg/ml Hoechst 333492 for 30 min at 37 °C. Cells were washed extensively with ice cold PBS pH 7.4.

Prior to imaging with the epifluorescent microscope, anti-fading solution (Daka Fluorescent Mounting Medium) was applied to prevent photo bleaching. Fluorescent images were captured by using the epifluorescent microscope (AxioVersion 4.5. Apotome software, Carl Ziess) EC plan-Neofluar 20x and 40x/1.30 Oil Dic M27 objective, with DAPI, FITC, and Texas Red filters. For Z-stack images, 0.5 μm thicknesses of Z-axis planes were taken and the 3D images were generated and analysed.

### Drug treatment

**Fig. 1:**
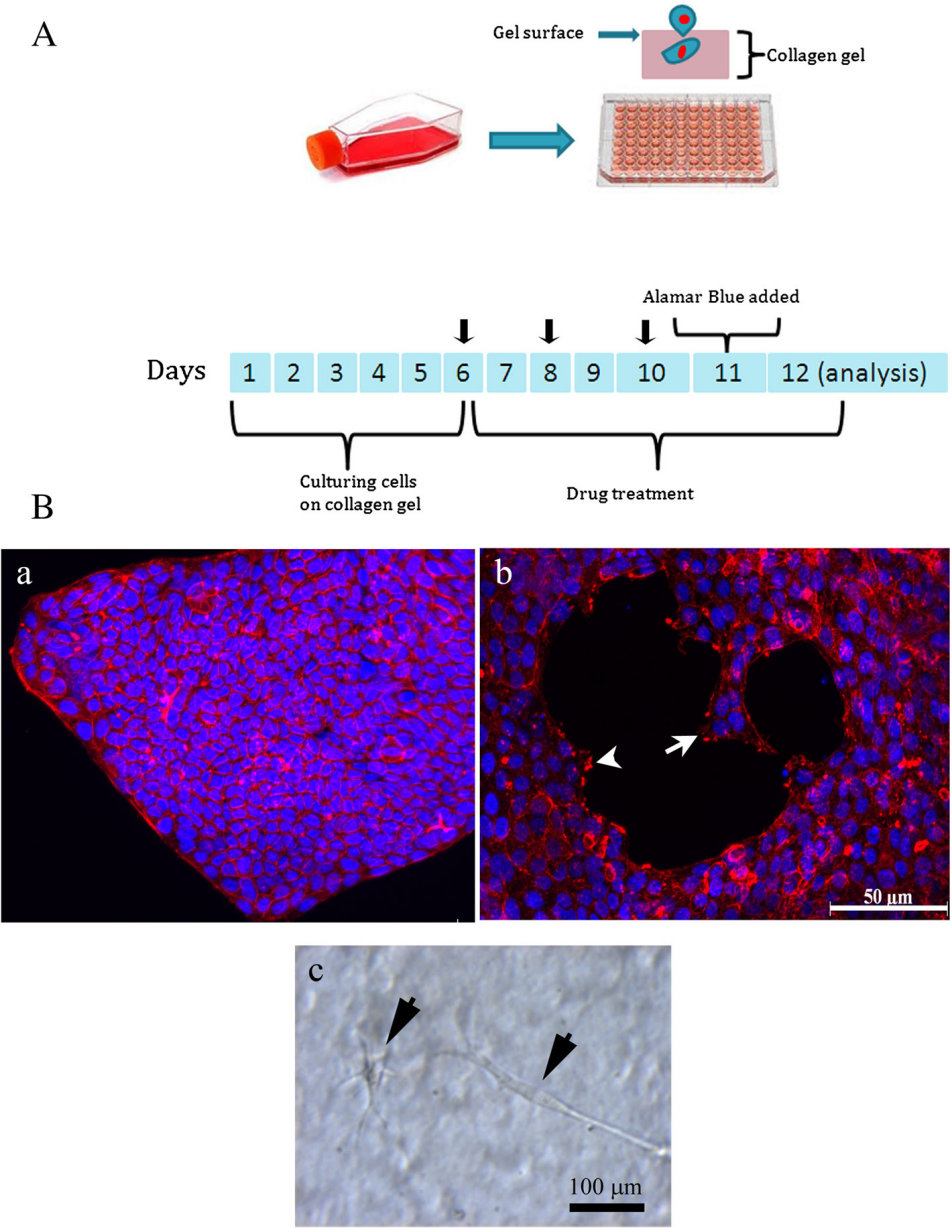
Experimental setup and growth of ovarian cancer cells cultured on top of collagen gels in a 96-well plate. **a**. Two cell lines, SKOV-3 and OVCAR-5, were cultured on the top of collagen gel overlaid 96 well plates for 6 days and treatment with active compounds for 6 days. On day 11, Alamar blue was added for overnight and the change of colour as the indication of cellular metabolism was examined on day 12. **b** OVCAR-5 cells grew on top of the gel as a compact sheet without the projection of filopodia suggesting this cell line is non invasive cancer cells (**a**). SKOV-3 cells showed the formation of filopodia at the invasive front of cells (**b**, arrow) and there was an appearance of actin bundles accumulation of invasive cells (**b**, arrow head). The invasive SKOV-3 cells displayed mesenchylmal morphological appearance (**c**, arrows). Red is actin staining and blue is the nucleus

As shown in Fig. 1a, at day 6 (as indicated arrow) the total volume of media in each well was 200 μl. Prior to the addition of fresh media supplemented with active compounds, 100 μl of media were removed from each well, and 100 μl of fresh media containing active compounds at concentrations of 10 μM resveratrol, 10 μM EGCG, 0.2 μM paclitaxel, 2 μM Everolimus, and 20 μM cisplatin were added. Therefore, the final concentrations of active compounds were 5 μM for resveratrol and EGCG, 0.1 μM for paclitaxel, 10 μM cisplatin, and 1 μM everolimus, Again at day 8 and day 10 (as indicated arrow), 100 μl of cell media were removed from each well and 100 μl of fresh media containing 5, 0.1, 10, 1 μM of resveratrol and EGCG, paclitaxel, cisplatin, and everolimus were added, respectively. Alamar Blue dye solution (20 μl) was added to each well on day 11 and incubated with cells for 24 h before analysis on day 12. The absorbance at each well of the 96-well plate was read at 560 and 600 nm using a microplate reader (Varioskan Flash®, Thermo Scientific)). The stock solutions of paclitaxel (Sigma, New Zealand), cisplatin (Sigma, New Zealand), and everolimus (LC Laboratories, MA, USA) were made in 100 % dimethyl sulfoxide (DMSO). Resveratrol (Sigma, New Zealand) stock solution was made in 50 % DMSO: 50 % PBS pH 7.4. The stock solution of EGCG (Sigma, New Zealand) was dissolved in PBS pH 7.4. Controls incubations contained similar concentration to that of DMSO in the highest dose of treated samples.

### ELISA of vascular endothelial growth factor (VEGF)

ELISA of VEGF was performed using the DuoSet Human VEGF ELISA Kit (R&D System) that detectsVEGF-A isoforms. Cell media from at least four independent experiments were collected and analysed for VEGF.

### Statistical analysis

Cellular metabolism was calculated from the difference of absorbance at 600 and 570 nm. Data represented at least four independent experiments. The mean and standard error were reported and significant changes (*p* < 0.05) were determined by student’s *t*-tests.

## Results

As shown in Fig. 1a, we cultured ovarian cancer cell lines, SKOV-3 and OVCAR-5, on the top of polymerised collagen gel for 6 days prior to challenging them with cytotoxic drugs (paclitaxel and cisplatin), a targeted drug (everolimus) and food compounds (resveratrol and EGCG). As indicated in Fig. 1b, after six days of culture, OVCAR-5 cell line (Fig. 1b-a) grew on top of the collagen gel and formed a compact sheet, and small compact colonies (data not shown) were also observed. The staining of actin was restricted to the region of cell-cell contacts. At the protrusive front of OVCAR-5, the cell sheet exhibited a lack of filopodia formation suggesting the cells were not competent to invade the collagen gel. On the other hand, actin filaments were observed at the leading edge of SKOV-3 cells. There were notable formations of filopodia (arrow Fig. 1b-b) and the accumulation of actin bundles of cells at the leading front (arrow head Fig. 1b-b). Occasionally, there were some invasive SKOV-3 cells observed below the surface of the gel (Fig. 1b-c). These invasive SKOV-3 cells displayed an elongated morphological appearance similar to mesenchymal morphology (Fig. 1b-c, arrows). On the contrary, OVCAR-5 cell line grew only on the top of collagen gel surface without any sign of invasion. Our result using OVCAR-5 was consistent with a previous observation [11].

**Fig. 2:**
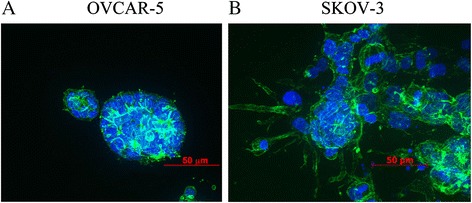
Morphological characteristics of OVCAR-5 and SKOV-3 cultured inside the collagen gel. OVCAR-5 formed spherical colony without the invasive capability when it cultured inside collagen gels (**a**) but the colony of SKOV-3 showed invasive capacity at the edge of colony that produced the intrusion of the plasma membrane, indicating invasive behaviour (**b**). Actin staining was shown in green and the nucleus was shown in blue

To further confirm that the cell lines have different invasive capacity when they were exposed to collagen gels, we embedded single cell suspensions of the two cell lines inside collagen gels and allowed them to grow for 6 days. Again a difference was observed. OVCAR-5 cell line grew as compact spherical colonies without the migration of cells at the edge of the colony (Fig. 2a). In contrast, SKOV3 cell line at the rim of the colony exhibited an invasive characteristic by producing projection of filopodia (Fig. 2b).

**Fig. 3:**
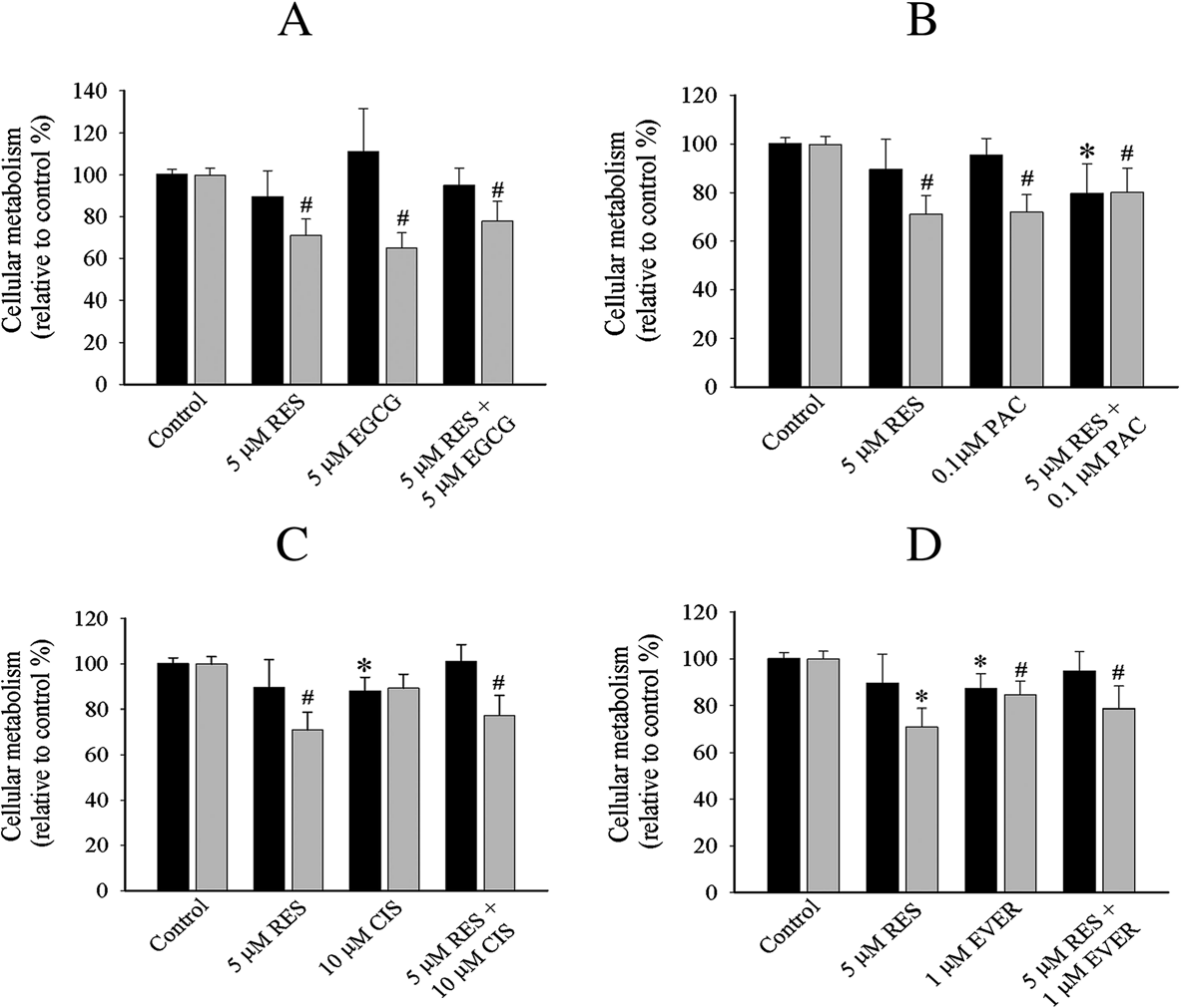
Cellular metabolism profiles of OVCAR-5 cell line with single and combination treatment of resveratrol + EGCG (**a**), resveratrol + paclitaxel (**b**), resveratrol + cisplatin (**c**), resveratrol + everolimus (**d**) in 2D cell monolayers (black bar) and 3D ECM (grey bar). The representative graph in 2D cell monolayers and 3D ECM was the relative value to the control. The statistical difference of single and combination in 2D cell monolayers (* *P < 0.05, student’s t-test*) and 3D ECM *(* ^***#***^ *P < 0.05, student’s t-test*) was compared between the control and treated cells. Data was obtained from at least four independent experiments with triplicate

**Fig. 4:**
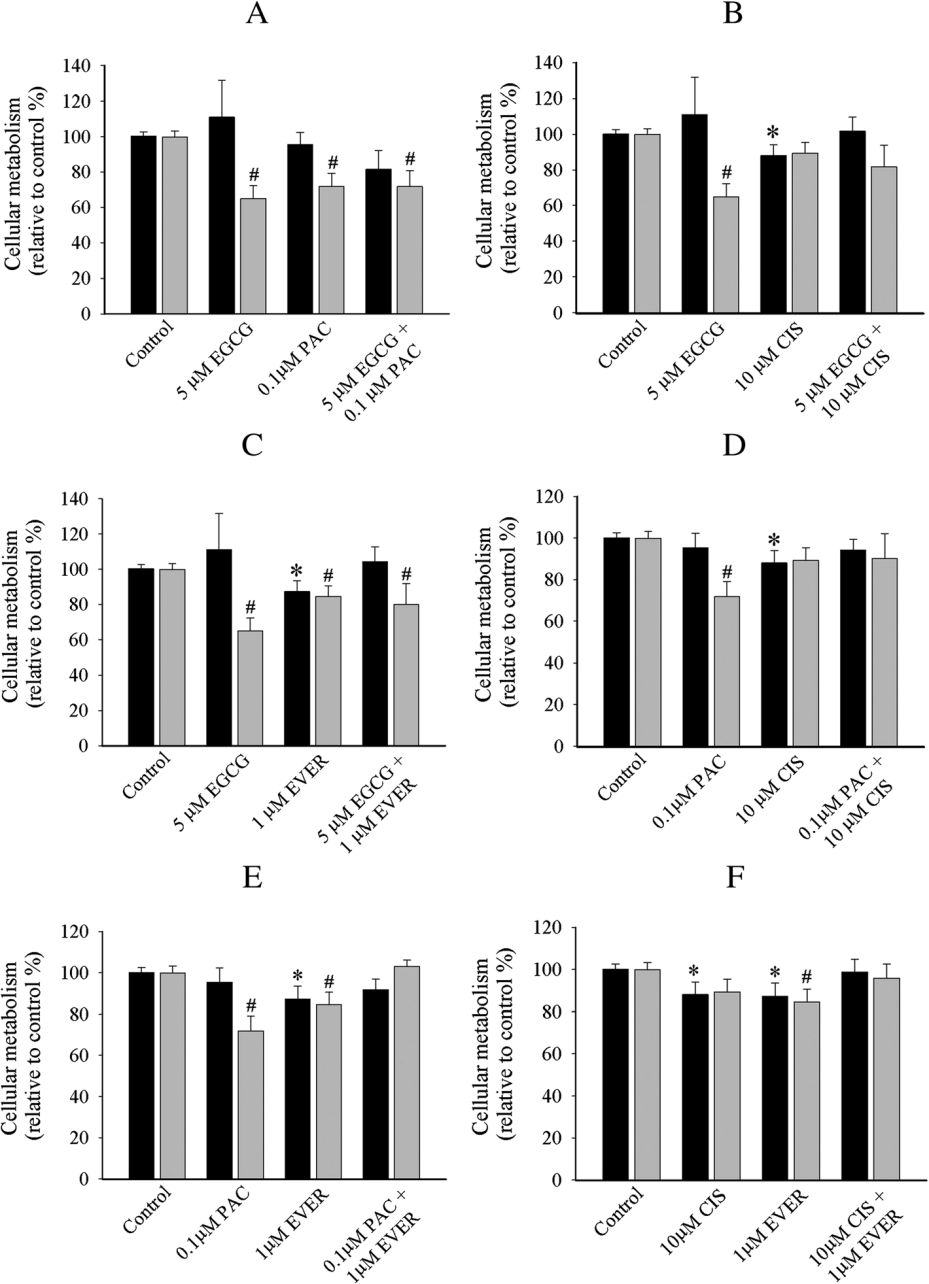
Cellular metabolism profiles of OVCAR-5 cell line with single and combination treatment of EGCG + paclitaxel (**a**), EGCG + cisplatin (**b**), EGCG + everolimus (**c**), paclitaxel + cisplatin (**d**) paclitaxel + everolimus (**e**) and cisplatin + everolimus (**f**) in 2D cell monolayers (black bar) and 3D ECM (grey bar). The representative graph in 2D cell monolayers and 3D ECM was the relative value to the control. The statistical difference of single and combination in 2D cell monolayers (* *P < 0.05, student’s t-test*) and 3D ECM *(* ^***#***^ *P < 0.05, student’s t-test*) was compared between the control and treated cells. Data was obtained from at least four independent experiments with triplicate

Next, we determined the efficacy of single drug and combination treatments in cell monolayers and cells on top of collagen gels. Single and combination treatments were selectively affected in OVCAR-5 cells in collagen gel (Figs. 3 and 4). Single treatments that reduced cellular metabolism only in collagen gel compared to the control included resveratrol (29 %, Fig. 3a), EGCG (35 %, Fig. 3a), and paclitaxel (28.1 %, Fig. 3b). Everolimus (Fig. 3d) reduced cellular metabolism in both cell monolayers (13 %) and collagen gel (16 %). The cell monolayers of OVCAR-5 cell line did not show any marked reduction of cellular metabolism except cisplatin (12 %, Fig. 3c).

The combinatory treatments were selectively affected in collagen gel OVCAR-5 cells. We categorised the action of these drugs into three modes of action. First, the combinations that exerted a marked reduction of cellular metabolism only in collagen gels included resveratrol + EGCG (22.2 %, Fig. 3a), resveratrol + cisplatin (23 %, Fig. 3c), resveratrol + everolimus (21 %, Fig. 3d), EGCG + paclitaxel (29 %, Fig. 4a), and EGCG + everolimus (20 %, Fig. 4c). Second, the combination that showed anti-metabolic activity in both 2D monolayers (20.4 %) and collagen gels (20 %) was only resveratrol + paclitaxel (Fig. 3b). Third, the combinations that did not show any reductions in either monolayers or collagen gels were EGCG + cisplatin (Fig. 4b), paclitaxel + cisplatin (Fig. 4d), paclitaxel + everolimus (Fig. 4e) and cisplatin + everolimus (Fig. 4f). There was no additive or synergistic inhibition in the combination treatment. Overall, OVCAR-5 cell monolayers showed limited response to single and combination treatments.

**Fig. 5:**
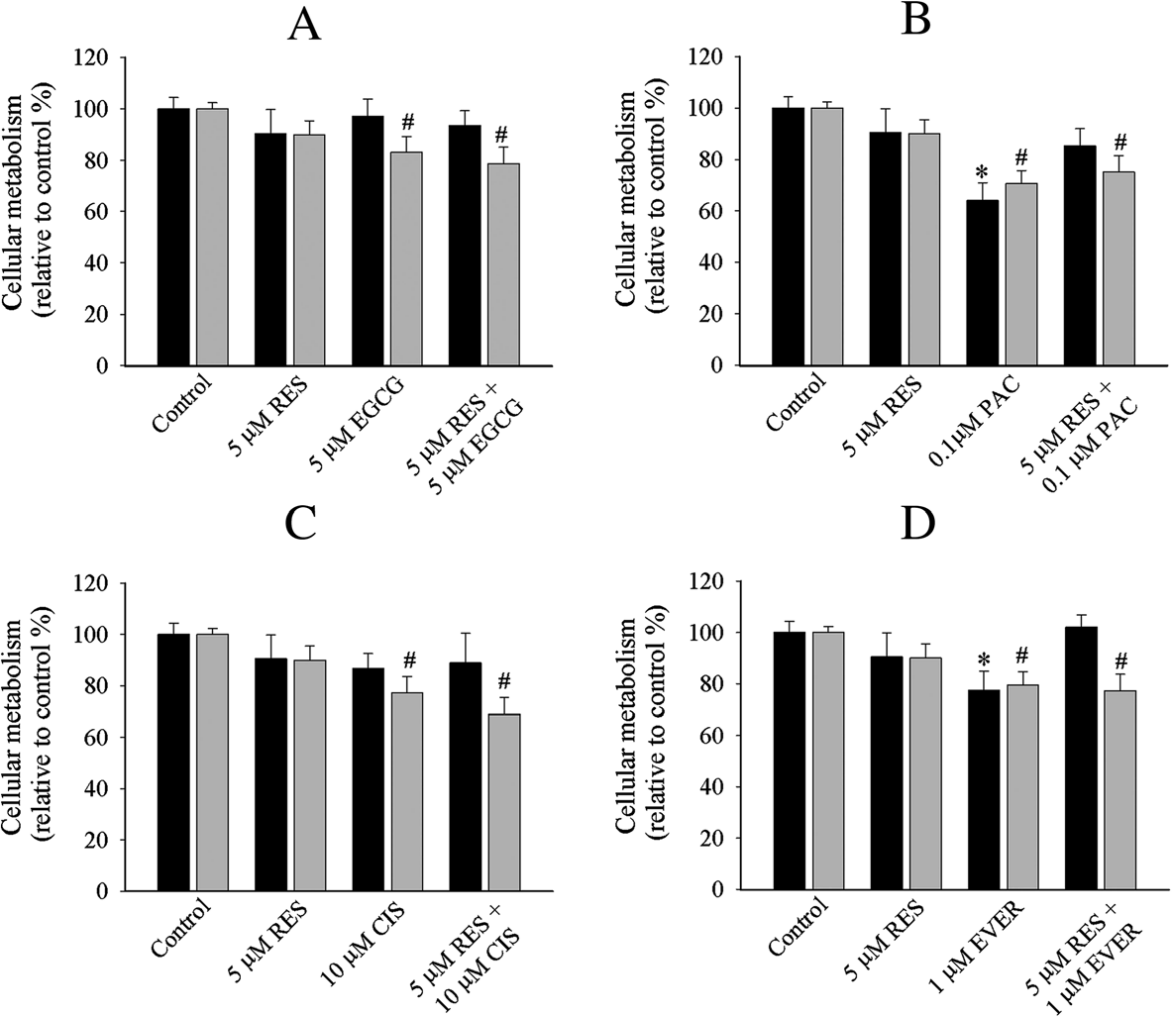
Cellular metabolism profiles of SKOV-3 cell line with single and combination treatment of resveratrol + EGCG (**a**), resveratrol + paclitaxel (**b**), resveratrol + cisplatin (**c**), resveratrol + everolimus (**d**) in 2D cell monolayers (black bar) and 3D ECM (grey bar). The representative graph in 2D cell monolayers and 3D ECM was the relative value to the control. The statistical difference of single and combination in 2D cell monolayers (* *P < 0.05, student’s t-test*) and 3D ECM *(* ^***#***^ *P < 0.05, student’s t-test*) was compared between the control and treated cells. Data was obtained from at least four independent experiments with triplicate

**Fig. 6:**
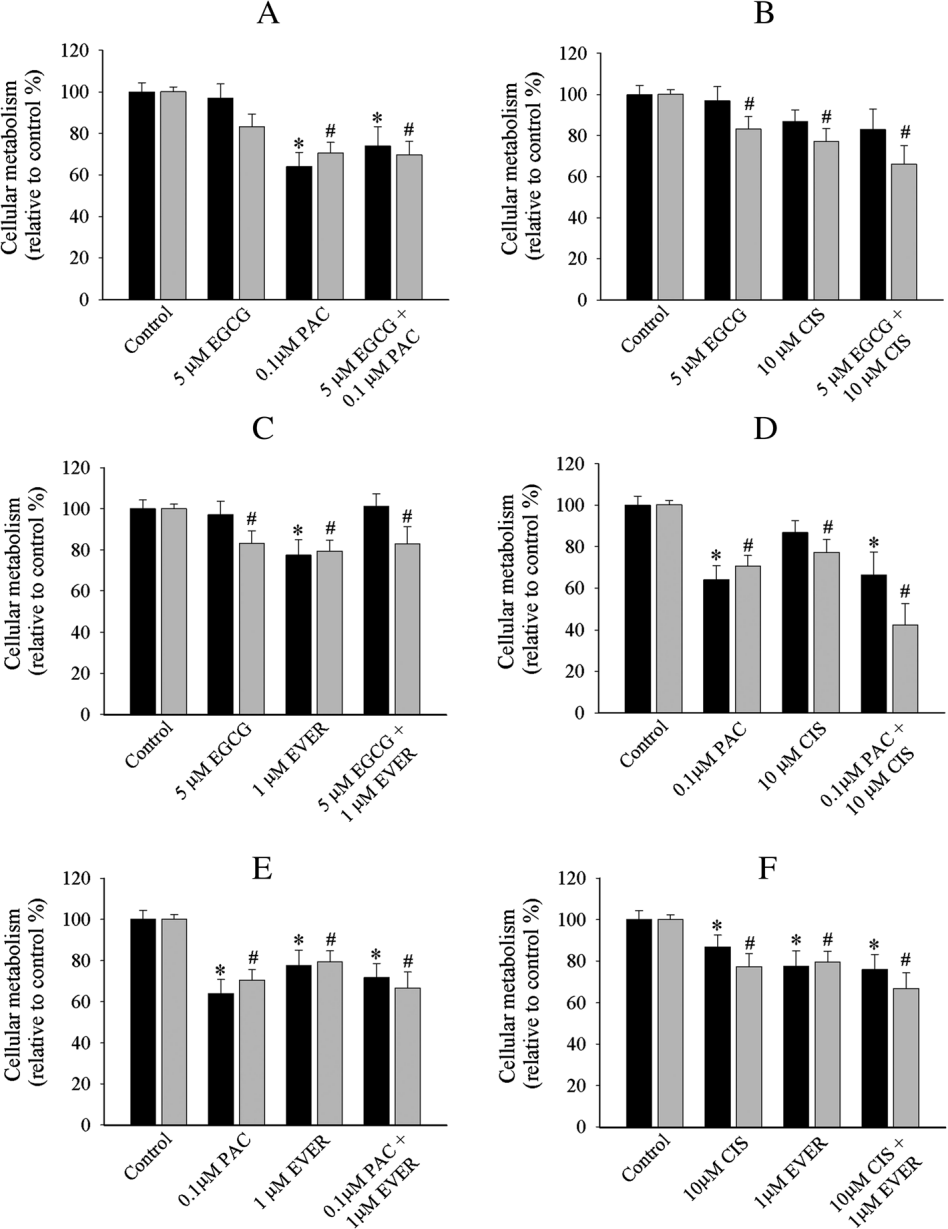
Cellular metabolism profiles of SKOV-3 cell line with single and combination treatment of EGCG + paclitaxel (**a**), EGCG + cisplatin (**b**), EGCG + everolimus (**c**), paclitaxel + cisplatin (**d**), paclitaxel + everolimus (**e**) and cisplatin + everolimus (**f**) in 2D cell monolayers (black bar) and 3D ECM (grey bar). The representative graph in 2D cell monolayers and 3D ECM was the relative value to the control. The statistical difference of single and combination in 2D cell monolayers (* *P < 0.05, student’s t-test*) and 3D ECM *(* ^***#***^ *P < 0.05, student’s t-test*) was compared between the control and treated cells. Data was obtained from at least four independent experiments with triplicate

The mono- and bi-combinatorial approaches of compounds were also conducted in cell monolayers and collagen gels of SKOV-3 cell line. First, the single treatments that significantly reduced cell metabolism only in collagen gels included EGCG (17 %, Fig. 5a) and cisplatin (23 %, Fig. 5c). Second, single treatments that reduced cellular metabolism in both cell monolayers and collagen gels were paclitaxel (36 % cell monolayers *vs* 30 % collagen, Fig. 5b) and everolimus (22 % cell monolayers *vs* 20 % collagen, Fig. 5d). Third, the combinations that reduced cellular metabolism only in collagen gels included resveratrol + EGCG (21 %, Fig. 5a), resveratrol + paclitaxel (25 %, Fig. 5b), resveratrol + cisplatin (31 %, Fig. 5c), resveratrol + everolimus (23 %, Fig. 5d), EGCG + cisplatin (34 %, Fig. 6b), and EGCG + everolimus (17 %, Fig. 6c). Finally, the combinations that reduced cell metabolisms in both cell monolayers and collagen gels included EGCG + paclitaxel (26 % cell monolayers *vs* 31 % collagen, Fig. 6a), paclitaxel + cisplatin (34 % cell monolayers *vs* 61 % collagen, Fig. 6d), paclitaxel + everolimus (28 % cell monolayers *vs* 33 % collagen, Fig. 6e), and cisplatin + everolimus (24 % cell monolayers *vs* 33 % collagen, Fig. 6f). Again, there was a lack of additive and synergistic inhibition of cellular metabolism in the combination treatments of SKOV-3 line.

**Fig. 7:**
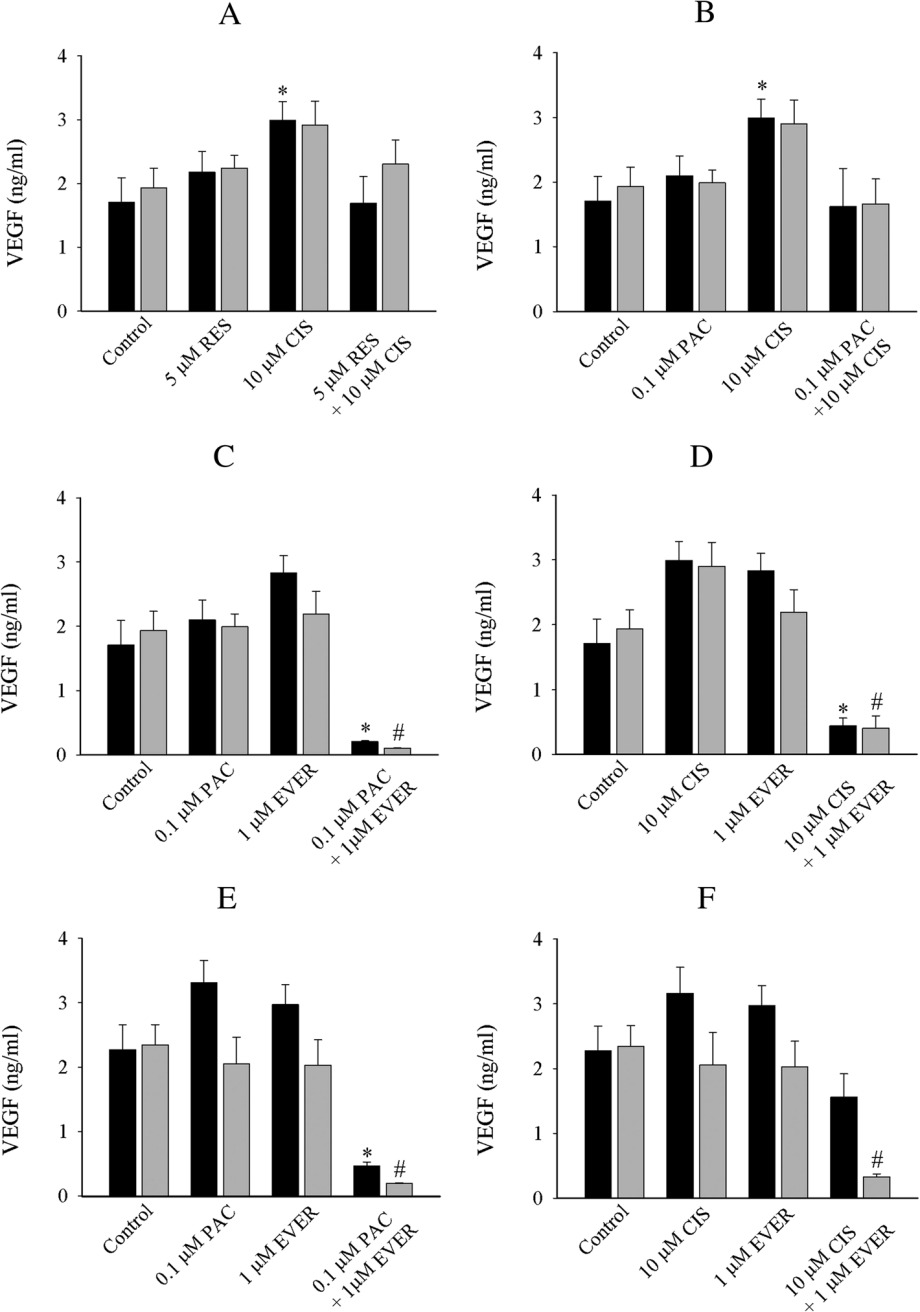
Production of secreted vascular endothelial growth factor (VEGF) of OVCAR-5 (**a**, **b**, **c**, and **d**) and SKOV-3 cells (**e** and **f**) in 2D cell monolayers (black bar) and 3D ECM (grey bar). The statistical difference of single and combination in 2D cell monolayers (* *P < 0.05, student’s t-test*) and 3D ECM *(* ^***#***^ *P < 0.05, student’s t-test*) was compared between the control and treated cells. The statistical difference of between 2D cell monolayers and 3D ECM are donated ** *(P < 0.05, student’s t-test*). Data was obtained from at least four independent experiments with triplicate

Next, we evaluated the production of secreted VEGF in the cell media after drug treatments. Single treatment of OVCAR-5 cell monolayers with cisplatin significantly increased the secreted VEGF (1.8 ng/ml control *vs* 3 ng/ml cisplatin, Fig. 7a). The combination of everolimus with paclitaxel (Fig. 7c) and cisplatin (Fig. 7d) reduced the VEGF secretion in both 2D cell monolayers and collagen gels. These combinations were also reproducible in SKOV-3 cell line (Fig. 7e, f). However, in SKOV-3 line the combination of everolimus with paclitaxel and cisplatin produced a greater significant reduction in collagen gels than 2D cell monolayers. Other combinations did not change the VEGF secretion in cell monolayers and collagen gels in both cell lines (data not shown).

## Discussion

We present a simple reproducible a 96-well collagen gel model for cell culture. The system is easy to set up, inexpensive, quick to perform, and suitable for high-throughput screening. The model provides an environment closely comparable to those experienced by ovarian cancer cells on the peritoneal membrane surface and the composition of the gel in our study is constituted to partly replicate the properties of the membrane. The model, therefore, enables us to study cell growth, survival, responsiveness to anti-cancer drugs and invasive characteristics at the early stage of tumorigenic progression at the peritoneal membrane lining.

The 96-well format may provide a convenient platform as a pre-clinical drug screening tool and for exploring biological pathways, which has not been reported previously for ovarian cancer. This system revealed that cells exhibit different drug sensitivities when cultured on traditional 2D monolayers or on the collagen gels and thus confirmed that the environments elicit distinct behaviours. Our project has not yet determined the influence of different gel compositions on ovarian cancer cell characteristics. We have used a murine collagen in this preparation, but the difference from human collagen is small as collagens are highly conservative proteins in vertebrates [12, 13] and the murine collagen is a well-established component of in vitro ECM studies [2, 3, 5, 14]. The compositions of ECM used in our study are closely similar to those present in the human peritoneal membrane surface [15]. The concentrations of collagen I, IV and laminins in our collagen model are consistent with previous studies [16, 17]. It is increasingly recognised that the different compositions and concentrations of ECM can contribute to the invasive phenotypes of cancer cells as previously described [17]. Our study does not yet determine the influence of various concentrations of collagens on an invasive phenotype of ovarian cancer cells; an increased stiffness of collagen compositions mimicking the interstitial collagen components has reported to facilitate invasive behaviour in breast cancer cells [17].

Drugs chosen in our study are categorised into cytotoxic agents (paclitaxel and cisplatin), targeted agent (everolimus) and bioactive food compounds (resveratrol and EGCG). The concentration of each compound was selected based on their bioavailability in plasma of humans. In cancer patients, the average peak plasma concentrations of paclitaxel after a 24 h infusion have ranged from 0.23-0.43 μM [18]. Cisplatin plasma concentration is about 2.6 μM in cancer patients after one hour IV [19]. Peak concentration of everolimus is approximately 0.1-5 μM [20]. After ingestion of 5 grams resveratrol and 1.6 grams of EGCG, the peak plasma concentration of native compounds after one hour reached approximately 5 μM and 7.4 μM, respectively [21]. Our results indicate the effect on cellular metabolism is drug- and cell culture format- dependent. Cells in 2D monolayers of OVCAR-5 and SKOV-3 show limited responses to single and combination drug treatments compared to collagen gels. In collagen gel model, there are six drug combinations that show a significant reduction of cell metabolism in OVCAR-5 line and ten drug combinations exhibit anti-metabolism in SKOV-3 line. Our study shows that the bi-combinatory drug approach does not have any additive or synergistic effect in comparison with the mono-treatment. The possible explanations for this unresponsiveness to the drug combinations would be the antagonistic interaction of drug combination that has been previously reported [22]. More importantly, the sequence of drug treatment may play an important role in additive/synergistic effects. Sequential anticancer drug administration is currently recognised as an important factor in cancer treatment [23]. In our study, both drugs are mixed at the beginning of treatment. In the clinic, co-administration of cisplatin and paclitaxel is introduced every three weeks for total of six cycles. A targeted drug including everolimus is normally used as adjuvant regimen. For our future study, we wish to use collagen gel model of ovarian cancer cells to investigate the various sequences of experimental drug combinations and then validate these combinations in an animal model.

Paclitaxel and cisplatin are cytotoxic drugs and commonly used in first-line treatment in women with advanced ovarian cancer. Paclitaxel is a tubulin stabilising agent that inhibits cancer cell division in the G2/M phase of the cell cycle and cell motility [24]. Our results show a greater reduction of cell metabolism in paclitaxel treated OVCAR-5 in collagen gel than cell monolayers. This may suggest that tubulin expression of cancer cells may be higher in collagen gels than cell monolayers. Proteomic analysis of liver cancer cells maintained in collagen gels reveals the increased level of several cytoskeletal associated proteins including tubulin [25]. Therefore, compromising cytoskeletal structure of cancer cells in collagen gel can produce a profound effect on cell viability. Cisplatin is a platinum based compound that crosses links with DNA and triggers apoptosis [26]. Platinum resistant ovarian cancer cells are highly prevalent, and the molecular basis of the resistance is attributed to several factors [27]. Both OVCAR-5 and SKOV-3 have been previously reported to be cisplatin resistant cell lines [28, 29]. Cisplatin has little effect on OVCAR-5 cells on collagen gel but shows a marginal inhibition in cell monolayers. On the contrary, cisplatin reduces cellular metabolism of SKOV-3 in the collagen gel model but not in cell monolayers. Therefore, responsiveness to cisplatin observed in our study is cell culture format- and cell type- dependent. Everolimus is a targeted drug that specifically inhibits the function of mammalian target of rapamycin (mTOR) pathway, which is dysfunctional in a subset of ovarian cancers and is associated with the up regulation of PTEN protein mutation and PI-3 K hyperactivation [30]. A previous study showed everolimus exhibits an anti-proliferative effect in cell monolayers and in in vivo animal model [6]. Our results show everolimus equally affects cell metabolisms in both cell monolayers and collagen gel model. The responsiveness of ovarian cancer cells on collagen gel to cytotoxic and targeted agents observed in this present study is consistent with other types of cancers in 3D models. For example, breast cancer cells are more sensitive to Her-2 and MEK inhibitors in 3D ECM than 2D cell monolayers [14, 31].

Our data show that physiological doses (5 μM) of resveratrol and EGCG can reduce cellular metabolism in collagen gels but not in cell monolayers. The field of research of resveratrol and EGCG and their potential anti-cancer properties in vitro has been active for decades. Resveratrol and EGCG are from grape and green tea, respectively, and are well known to display an array of anti-tumour activities in various types of cancer cell lines including ovarian cancer, but knowledge of their anti-tumour activities in ovarian cancer in vivo is very limited [32]. In addition, most of available studies of resveratrol and EGCG have been using non-physiological doses (50–100 μM) in cell monolayers. These high doses are not reached in plasma of animal models and human. This may raise a question of the suitability of cell monolayers for a pre-clinical drug screening and exploring biochemical pathways for cancer research because it is uncertain whether the drug efficacy produced in 2D cell monolayers is a true response. One possible explanation for high levels of resveratrol and EGCG being necessary in 2D cell monolayers is that the alteration of protein expression, which responds to such active compounds, may be insufficient in cell monolayers to detect readily, and therefore high doses are required to produce an observable effect. On the other hand, those protein expressions and functions may be fully optimised in a collagen gel and, therefore a lower amount of active compounds is needed to produce a similar significant effect.

It is important to note that the present study shows the invasive capacity of ovarian cancer cells on collagen gels, and also the invasion is notable when cells are inside the collagen gel and is cell line-dependent. SKOV-3 cell line has high expression of EGFR, Her-2, and c-Met proteins and negative E-cadherin [33, 34]. Our results are consistent with a previous study [35] suggesting SKOV-3 cell line exhibits invasive capacity when culturing on the top or within collagen gels. SKOV-3 cells shows aggressive growth, metastasis, and invasion in a mouse model [36]. OVCAR-5 cell line has low expression of EGFR and Her-2 but high expression of c-Met and E-cadherin [37]. Our results show that OVCAR-5 cell line does not display invasive capacity in collagen gels, suggesting EGFR/Her-2 oncogenic expression may be a primary factor in the aggressiveness of tumour invasion in 3D gels [38]. Over expression of EGFR and Her-2 correlate with invasive colorectal cancer cells in collagen gels [39]. There is a lack of in vitro cell models apart from 2D cell monolayers to assess invasive phenotypes of ovarian cancer cells. Therefore, our collagen gel model will be the forefront of biological tool that may provide a mean of rapid assessment of oncogenic molecule alteration that facilitates invasive potential in a subset of patients with advanced disease. This may lead us to devise tailored treatment based on the invasive characteristic of patient’s cancer cells. There are few reports studying the growth property of ovarian cancer cells in 3D collagen gel, but these studies have cultured ovarian cancer cell lines within a collagen gel or a synthetic matrix as a representative of in vivo primary and secondary growing tumour [40, 41]. Our system is, however, designed to replicate the early growth stage of attached and invasive ovarian cancer cells to the surface of the peritoneal membrane where cancer cells are not yet fully established secondary tumour nodules.

It is well known that secretion of VEGF is strongly stimulated by tumour micro-environmental conditions including hypoxia, an elevated expression of oncogenic proteins, and stress- induced treatment. All these modulators are observed in cancer patients. In our study, we could rule out hypoxic induced effects because the level of VEGF secreted from cells monolayers and collagen gels is not significantly different. However, the stress-related VEGF production induced by drugs may be possible. Even though the increase of VEGF in collagen gels is not significant compared to the control but the elevated VEGF is more pronounced in 2D cell monolayers than collagen gels with cisplatin treatment. It is worthy to note that paclitaxel and everolimus increase VEGF in 2D cell monolayers but not in collagen gels. It is unclear how collagen matrix can influence the production of angiogenic proteins altered by anti-cancer agents. Again, it is difficult to pinpoint the exact modulator that can influence VEGF responses in our study, but results may indicate that the production of VEGF in 2D cell monolayers and collagen gels is modulated by drugs in a different manner.

In summary, we demonstrated that ovarian cancer cells on the collagen gel in a 96-well format can be used to evaluate the cytotoxicity of anti-cancer drugs on growth of ovarian cancer cells and to study an invasive phenotype of ovarian cancer cells in a pre-clinical setting. The collagen gel model provides an approximate physiological microenvironment in which to study the early attachment of metastatic growth of ovarian cancer cells at the peritoneal membrane. The analysis of cellular metabolism in individual ovarian cancer cell lines acted as a preliminary guide to the cytotoxicity of anticancer agents. In a clinical setting, each patient tumour will have a distinct molecular background, and this collagen gel model may have potential to provide an important preclinical tool for patient related drug screening, for example to identify tumours with platinum resistant disease.
